# Complete Genome Sequence of the Avian Paramyxovirus Serotype 5 Strain APMV-5/budgerigar/Japan/TI/75

**DOI:** 10.1128/genomeA.01005-16

**Published:** 2016-09-22

**Authors:** Takahiro Hiono, Keita Matsuno, Kotaro Tuchiya, Zhifeng Lin, Masatoshi Okamatsu, Yoshihiro Sakoda

**Affiliations:** a Laboratory of Microbiology, Department of Disease Control, Graduate School of Veterinary Medicine, Hokkaido University, Sapporo, Hokkaido, Japan; b Global Station for Zoonosis Control, Global Institution for Collaborative Research and Education (GI-CoRE), Hokkaido University, Sapporo, Hokkaido, Japan; c Nippon Institute for Biological Science, Ome, Tokyo, Japan

## Abstract

Here, we report the complete genome sequence of the avian paramyxovirus serotype 5 strain APMV-5/budgerigar/Japan/TI/75, which was determined using the Illumina MiSeq platform. The determined sequence shares 97% homology and similar genetic features with the previously known genome sequence of avian paramyxovirus serotype 5 strain APMV-5/budgerigar/Japan/Kunitachi/74.

## GENOME ANNOUNCEMENT

In 1974, an acute fatal disease affecting caged budgerigars was reported in Kunitachi, Tokyo, Japan, and an avian paramyxovirus (APMV) classified in the fifth serogroup of APMV, namely, strain APMV-5/budgerigar/Japan/Kunitachi/74 (Kunitachi), was isolated from a dead budgerigar (1). In 1975, similar diseases affecting budgerigars were reported throughout Japan, and a paramyxovirus, strain APMV-5/budgerigar/Japan/TI/75 (TI), was isolated from a dead budgerigar (2). To date, there are only four reports in the world on APMV-5 infections (3, 4). However, genetic information of APMV-5 was available only for the Kunitachi strain. Here, we successfully obtained a complete genome sequence of the TI strain.

The TI strain was inoculated onto quail fibrosarcoma cell line (QT-6 cells). Four days after inoculation, a cytopathic effect with syncytia was observed and the supernatant was harvested. Viral RNA was extracted from the supernatant using the QIAamp viral RNA mini kit (Qiagen). With the extracted RNA, an MiSeq library indexed with i503 and i704 primers was prepared using the NEBNext Ultra RNA library prep kit and Multiplex Oligos for Illumina. Of the library, 0.16 fmol was pooled in a total of 9 fmol of MiSeq libraries comprising nonparamyxovirus libraries and sequenced on the MiSeq using the MiSeq reagent kit v3 (Illumina) with 2 × 300-bp paired-end read lengths. Reads were *de novo* assembled using CLC Genomic Workbench (CLC bio). The 3′-end and 5′-end of the virus genome were determined by the rapid amplification of the cDNA end (RACE) method and conventional Sanger sequencing as described previously (5), with virus-specific primer (VSP) 5-1 (5′ TCCAGTCATGGGTGGGCAAC 3′), VSP5-2 (5′ CGATCCTGAGAGTGTGATAGGAC 3′), VSP5-3 (5′ GGTCTTAGTAGATCAGCAACCC 3′), VSP3-1 (5′ GTTGCTGAATCGCTGACTATCC 3′), and VSP3-2 (5′ CTTGCGAGATAAGTCGGGAG 3′). The sequence was determined using the BigDye Terminator version 3.1 cycle sequencing kit (Thermo Fisher Scientific) and the 3500 Genetic Analyzer autosequencer (Thermo Fisher Scientific).

The complete genome sequence of the TI strain comprised 17,262 nucleotides. The genome nucleotide length is divisible by six, which, by the “rule of six,” confirms its membership in the family *Paramyxoviridae* (6). The genome organization of the TI strain was predicted as 3′- leader-N-P/V/W-M-F-HN-L-trailer-5′, which is similar to that of the Kunitachi strain and other APMVs (7). The complete genome sequence of the TI strain shares 97% overall homology with that of the Kunitachi strain (accession no. GU206351). Amino acid sequence homology of each protein is 99% (N), 96% (P), 94% (V), 93% (W), 98% (M), 98% (F), 97% (HN), and 99% (L). The present results demonstrate that the TI strain shows high genetic similarity to the Kunitachi strain. Although APMV-5 caused a lethal disease in budgerigars, little is known about this pathogen. Virogenic avian paramyxovirus infection is important and needs to be differentiated from highly pathogenic avian influenza. Thus, the present results provide significant genetic information for the control of avian infectious diseases.

### Accession number(s).

The complete genome sequence has been deposited in DDBJ/EMBL/GenBank under the accession number LC168750. This paper describes the first version of the sequence.
